# Heat stress affects dairy cow health status through blood oxygen availability

**DOI:** 10.1186/s40104-023-00915-3

**Published:** 2023-09-02

**Authors:** Jia Zeng, Jie Cai, Diming Wang, Hongyun Liu, Huizeng Sun, Jianxin Liu

**Affiliations:** 1 Key Laboratory of Dairy Cow Genetic Improvement and Milk Quality Research of Zhejiang Province, College of Animal Sciences, Zhejiang University, Hangzhou, China; 2 Ministry of Education Key Laboratory of Molecular Animal Nutrition, Zhejiang University, Hangzhou, China

**Keywords:** Dairy cow, Health status, Heat stress, Lactation performance, Oxygen metabolism

## Abstract

**Background:**

Rises in global warming and extreme weather occurrence make the risk of heat stress (HS) induced by high ambient temperatures more likely in high-yielding dairy cows, resulting in low milk quality and yield. In animals, oxygen is involved in many physiological and metabolic processes, but the effects of HS on oxygen metabolism remain unclear. Thus, the current study aimed to investigate how oxygen metabolism plays a role in health status of dairy cows by measuring the milk yield, milk composition, and blood biochemical variables of cows under different levels of HS: none (No-HS), mild (Mild-HS), and moderate HS (Mod-HS).

**Results:**

The HS significantly increased rectal temperature (*P*_*treat*_ < 0.01) and respiration rate (*P*_*treat*_ < 0.01). Under Mod-HS, greater Na^+^ (*P* < 0.05) and lower total CO_2_, and pH (*P* < 0.05) were observed relative to those under No-HS and Mild-HS. Oxygen concentrations in both coccygeal artery and mammary vein (*P*_*treat*_ < 0.01) were lower under Mod-HS than under No-HS. Coccygeal vein concentrations of heat shock protein 90 (HSP90) (*P* < 0.05) increased during Mod-HS compared with those in cows under No-HS. Malondialdehyde increased during Mod-HS, and glutathione peroxidase (*P* < 0.01) increased during Mild-HS. Coccygeal vein concentrations of vascular endothelial growth factor (*P* < 0.01), heme oxygenase-1 (*P* < 0.01), and hypoxia-inducible factor 1α (*P* < 0.01) were greater in cows under Mod-HS than those under No-HS. Red blood cell count (*P* < 0.01) and hemoglobin concentration (*P* < 0.01) were lower in the coccygeal vein of dairy cows under Mild- and Mod-HS than those of cows under No-HS.

**Conclusions:**

Exposure to HS negatively impacts the health status and lactation performance of dairy cows by limiting oxygen metabolism and transportation. However, the specific mechanism by which HS affects mammary function in cows remains unclear and requires further exploration.

**Supplementary Information:**

The online version contains supplementary material available at 10.1186/s40104-023-00915-3.

## Introduction

Global warming and heat stress (HS) significantly affect livestock production. The HS reduces milk production in mid-lactation cows by 30%–40% [1, 2]. Reduced feed intake accounts for approximately half of total milk production decrease [3]. The decline in milk production induced by HS could potentially be attributed to an array of complex physiological alterations within the bovine body. These may encompass the apoptosis of mammary epithelial cells [4], modifications within the rumen microbiome [5], onset of systemic oxidative stress [6], and shifts in the overall health status of the dairy cow. However, the HS-induced physiological mechanisms associated with reduced milk synthesis and feed intake are not well understood.

The HS stimulates the production of reactive oxygen species (ROS), which are by-products of oxygen metabolism originating from cellular respiration. Oxidative stress occurs when there is an imbalance between ROS production and antioxidative defenses. Heat-stressed cows suffer from oxidative stress, which affects body substance metabolism and alters lactation performance [7, 8]. Previous research has focused on the effects of HS on the cow behavior and physiology [9, 10]. On the other hand, oxygen is involved in several important physiological and metabolic processes in animals, including ROS production and oxidative stress. Oxygen metabolism includes gas exchange, transport, and utilization, of which availability of oxygen is a complex biological process. Blood oxygen plays a vital role in metabolism and physiological regulation within the animal body. For example, high-yielding dairy goats have greater blood oxygen partial pressure and circulating oxygen availability than low-yielding goats [11]. Hemoglobin (HGB) is an iron-containing metalloprotein in red blood cells (RBC), and the main carrier of oxygen [12]. However, oxygen metabolism and availability in dairy ruminants under HS conditions are often overlooked.

Under HS conditions, metabolic processes in these tissues may be altered, potentially leading to changes in oxygen consumption, and consequently differences in oxygen concentrations in the venous blood. Furthermore, HS may influence blood flow and vascular function, which could also contribute to the differences in oxygen concentration. Therefore, we hypothesized that environmental HS would affect lactation performance in dairy cows by influencing oxygen metabolism. Previous studies have been usually conducted using a chamber to simulate HS or compare differences in cows’ physiology and metabolism between winter and summer [8, 13, 14]. The underlying mechanisms by which HS influences lactation performance and oxygen availability in dairy cows under natural conditions are largely unknown. In addition, few studies have monitored changes in cow physiology and oxygen metabolism in the same individual consistently under natural HS. Therefore, a self-controlled experiment was designed to elucidate HS effects on dairy cow health status from the perspectives of oxygen metabolism and oxygen availability. Our study provides novel insights into the physiological effects of HS on animals, which will help inform the development of strategies to alleviate HS in dairy cows.

## Materials and methods

### Experimental design and animal management

All experimental procedures were approved by the Animal Care Committee of the Zhejiang University (Hangzhou, China). Eighteen high-yielding Chinese Holstein cows (milk yield = 41.4 ± 0.47 kg/d, days in milk = 207 ± 4.2 d, parity = 2–3, mean ± standard error) were selected and housed within the same barn in a dairy farm. They were subjected to three conditions of varying HS intensity: no HS with a temperature-humidity index (THI) below 68 (No-HS, from May 14 to May 21), mild HS (Mild-HS, from May 22 to June 18, 68 ≤ THI ≤ 79), or moderate HS (Mod-HS, from June 21 to July 14, 79 < THI ≤ 88) [15] in their natural environment (Additional file 1: Fig. S1a). All the cows were observed over a two-month period, and samples were collected from these cows in all three conditions (Additional file 1: Fig. S1b).

Cows were fed three times per day with free access to water and housed in a shaded and sand-bedded free-stall barn equipped with waterers and fans. The natural environment and barn conditions were similar to those described previously [16]. Air circulation system was utilized, calibrated to operate at a fan speed of 20,000 m^3^/h. A precision sprinkler system was adopted for the cows, with sprinkler flow rate of 1.8 L/h. All the cows were fed with a total mixed ration formulated to meet their nutritional requirements [17] throughout the experiment. Basal diet ingredients and chemical composition are presented in Table S1.

During the experiment, multi-point observations were made to record the temperature and humidity of the farm using automatic temperature and humidity recorders with an accuracy of ± 0.2 °C and ± 2% relative humidity (RH) (TH20R; Shenzhen Huahanwei Technology Co., Ltd., Shenzhen, China). Five automatic temperature and humidity recorders, suspended at a height of about 2.0 m from the ground, were placed in the center of the barn and several locations around the barn. The THI was calculated based on the equation recommended by Dikmen and Hansen [18]: THI = (1.8 × T + 32) – [(0.55 – 0.0055 × RH) × (1.8 × T – 26)], where T = ambient temperature (°C) and RH = relative humidity (%). The THI values for different sampling days are shown in Fig. S1c.

### Lactation performance and physiological measurements

All cows were milked three times daily at 0500, 1400, and 2000. Milk production was recorded for three consecutive days and milk yield was collected using the APOLLO Milking System (GEA Farm Technologies, Naperville, USA). The collected milk samples were premixed at a ratio of 4:3:3 in a 50-mL centrifuge tube for later analysis. Milk composition was analyzed for protein, fat, lactose, milk urea nitrogen, and somatic cell count by infrared analysis [19] using a spectrophotometer (Foss-4000; Foss Electric A/S, Hillerod, Denmark).

Respiratory rate (RR) and rectal temperatures (RT) were measured for all cows at 0800, 1400, and 2000 during sampling days. The RR was calculated by taking mean cow total flank movements over two 60-s periods. The RT was measured with a digital thermometer (GLA M900, accuracy ± 0.1 °C, GLA Agricultural Electronics, CA, USA). Each cow was measured twice and the two values were averaged. The RR and RT of dairy cows at three different time points are shown in Table S2.

### Plasma metabolites and blood gas parameters

Blood samples were collected using BD Vacutainer anticoagulant lithium heparin vacuum tubes and blood collection needles, each of which was individually packaged and sterilized with cyclohexane prior to use. Samples were collected from the coccygeal vein, coccygeal artery, and mammary vein 3 h after morning feeding in 10-mL lithium heparin tubes. Two hundred microliters of all the blood samples were immediately used to measure blood gas parameters. The coccygeal vein samples were centrifuged at 3,000 × *g* for 15 min at 4 °C to obtain plasma for further analysis of hematological parameters and variable related to HS, hypoxia stress, and oxidative stress.

Blood oxygen concentration was measured using an i-STAT portable clinical analyzer (Heska Corporation, Loveland, CO, USA). Each i-STAT blood gas card came with a calibration solution for calibration. Determined blood gas parameters included pH; concentrations of sodium (Na^+^), potassium (K^+^), total carbon dioxide (CO_2_), ionized calcium (iCa^2+^); HGB; partial pressure of oxygen (pO_2_) and of carbon dioxide (pCO_2_); HCO_3_^−^; base excess in the extracellular fluid (BEecf); and oxygen saturation (sO_2_). An i-STAT CG8 + cartridge (Abbott Medical, Canada) was used as the blood gas card in the analyzer. Hematological parameters were determined using an automatic hematology analyzer (Automatic blood cell analyzer, Mindray B2600, Shenzhen, China).

### Heat stress-, hypoxia stress- and oxidative stress-related parameters

Commercial ELISA kits developed by Nanjing Jiancheng Bioengineering Institute (Nanjing, China) were used to analyze HS-related parameters in coccygeal vein plasma, including heat shock transcription factor (HSF; #H612), heat shock protein 70 (HSP70; #H264-2), HSP90 (#H264-3), and HSP27 (#H264-4).

Hypoxia-inducible factor 1α (HIF-1α; #H307-2), nitric oxide (NO; #A012-1-2), inducible nitric oxide synthase (iNOS; #H372-1), endothelial nitric oxide synthase (eNOS; #H195), heme oxygenase 1 (HO-1; #H246-1), and vascular endothelial growth factor (VEGF; #H044-2) levels were determined using a radial immunodiffusion assay (commercial kits provided by Nanjing Jiancheng Bioengineering Institute, Nanjing, China). Glutathione peroxidase (GSH-Px; #A005-1-2), malondialdehyde (MDA; #A003-1-2), total antioxidant capacity (T-AOC; #A015-3-1), and superoxide dismutase (SOD; #A001-1-2) were determined using a commercial kit (Nanjing Jiancheng Bioengineering Institute, Nanjing, China) according to previously reported procedures [20, 21]. Blood physio-biochemical analysis was performed using a 7020 Clinical Analyzer (Hitachi High-Tech Corporation). Total bilirubin concentration (#B-2014) was measured using commercial kits (Shanghai Juchuang Biotechnology Co., Ltd., Shanghai, China).

### Calculations and statistical analysis

Blood oxygen concentration was calculated using the following equation [22, 23]: oxygen concentration (%) = 0.003 × pO_2_ + 1.34 × HGB × sO_2_, where pO_2_ is the partial pressure of oxygen (mmHg), HGB is hemoglobin (g/dL), and sO_2_ is oxygen saturation (%). The arterio-venous difference (AVD) was calculated according to the method reported previously [24].

Statistical analysis was performed using SAS Analytics Software 9.4 (SAS Institute Inc., Cary, NC, USA), and differences among treatments were analyzed using orthogonal polynomial comparisons of linear and quadratic effects, with treatment as fixed variables and individual cows as random variables. Statistical model as follows:

1 $${y}_{ij}=\mu +{\tau }_{i}{ + \delta }_{j}+{L}_{i}+{Q}_{i}+{\epsilon }_{ij}$$

where ${y}_{ij}$ is the dependent variable of cow *j* in different HS treatment *i*, $\mu$ is the overall mean, ${\tau }_{i}$ is the fixed effect of different HS treatment, ${\delta }_{j}$ is the random effect of individuals that is assumed to follow normal distribution $N(0, I{\sigma }^{2})$, and $I$ is the identity matrix, ${L}_{i}$ is the linear effect of HS treatment *i*, ${Q}_{i}$ is the quadratic effect of HS treatment *i*, and ${\epsilon }_{ij}$ is the random residuals. The *P* values for treatment, linear, and quadratic effects were calculated and denoted as *P*_*trea*t_, *P*_*linear*_, *P*_*quadratic*_, respectively. The interactions between the levels of the fixed factors were evaluated by means of pairwise comparisons. Statistical comparisons among groups were carried out through a one-way analysis of variance (ANOVA), supplemented by Tukey's multiple comparisons post-hoc test using GraphPad Prism v8.0 (GraphPad Software, Inc.). Pearson’s correlation coefficient analysis was conducted to determine correlations between variables. The correlation analysis was performed using the “corrplot” package in R (https://www.r-project.org) [25, 26]. Results are presented as the mean ± standard error of the mean (SEM). A statistically significant difference was defined as *P* < 0.05 and highly significant at *P* < 0.01.

## Results

### Physiological measurements and lactation performance

Table 1 presents the changes in dairy cow physiological measurements and lactation performance. The HS increased the RT and RR of dairy cows (*P*_*treat*_ < 0.01). Milk yield and percentage of milk fat decreased (*P*_*treat*_ < 0.01) during the HS period, whereas somatic cell counts increased in cows under Mod-HS (*P*_*treat*_ < 0.01).

**Table 1:** Effects of heat stress on lactation performance of dairy cows

Item^1^	Heat stress			SEM	*P*-value		
	No	Mild	Moderate		Treat	Linear	Quadratic
Rectal temperature, °C	38.4^c^	38.8^b^	39.7^a^	0.08	< 0.01	< 0.01	< 0.01
Respiratory rate, bpm	43.4^c^	49.2^b^	76.0^a^	2.26	< 0.01	< 0.01	< 0.01
Yield, kg/d							
Milk yield	41.4^a^	36.8^b^	26.8^c^	0.70	< 0.01	< 0.01	< 0.01
ECM	49.6^a^	35.5^b^	26.0^c^	1.47	< 0.01	< 0.01	0.22
4%FCM	46.1^a^	31.1^b^	23.1^c^	1.53	< 0.01	< 0.01	0.07
Milk composition, %							
Fat	4.02^a^	2.88^b^	2.65^b^	0.28	< 0.01	< 0.01	< 0.01
Protein	3.51^a^	3.20^b^	3.04^b^	0.05	< 0.01	< 0.01	< 0.01
Lactose	5.08^a^	5.00^a^	4.73^b^	0.09	< 0.01	< 0.01	0.24
SCC, × 10^3^/mL	67.9^b^	93.3^b^	166.0^a^	25.96	< 0.01	< 0.01	0.30
MUN, mg/dL	18.1^a^	12.5^b^	14.4^b^	0.80	< 0.01	< 0.01	< 0.01

^1^*ECM* Energy-corrected milk yield, ECM = 0.3246 × milk yield + 13.86 × milk fat yield + 7.04 × milk protein yield, *FCM* Fat corrected milk yield, 4%FCM = 0.4 × milk yield + 15 × milk fat yield, *SCC* Somatic cell counts, *MUN* Milk urea nitrogen

^a–c^Means within the same row with different superscripts differ (*P* < 0.05)

### Blood gas profiles and oxygen concentration

Gas profiles in coccygeal vein of dairy cows are shown in Table 2. Under Mild-HS, the concentration of ionized Ca^2+^ was greater, whereas the concentrations of Na^+^ and K^+^ were lower than those under No-HS (*P* < 0.05). Under Mod-HS, greater Na^+^ (*P* < 0.05) and lower total CO_2_, pH, and BEecf (*P* < 0.05) were observed relative to those under No-HS and Mild-HS. No significant differences (*P*_*treat*_ > 0.05) were identified in the sO_2_ and pO_2_ among different HS conditions.

**Table 2:** Blood gas parameters in coccygeal vein of dairy cows under varying heat stress

Item^**1**^	Heat stress			SEM	*P*-value		
	No	Mild	Moderate		Treat	Linear	Quadratic
iCa^2+^, mmol/L	1.20^b^	1.93^a^	1.21^b^	0.06	< 0.01	0.91	< 0.01
Na^+^, mmol/L	136^b^	134^c^	138^a^	0.36	< 0.01	< 0.01	< 0.01
K^+^, mmol/L	4.46^a^	4.09^b^	4.14^b^	0.06	< 0.01	< 0.01	< 0.01
pO_2_, mmHg	33.7	40.1	37.2	3.70	0.48	0.50	0.32
sO_2_, %	64.3	68.8	66.3	2.75	0.48	0.59	0.28
Total CO_2_, mmol/L	30.7^a^	31.0^a^	27.2^b^	0.54	< 0.01	< 0.01	< 0.01
pCO_2_, mmHg	46.0	43.3	44.2	1.84	0.52	0.45	0.39
HCO_3_^−^, mmol/L	29.3	28.0	25.8	1.08	0.07	0.02	0.72
BEecf, mmol/L	4.61^a^	5.33^a^	0.56^b^	0.56	< 0.01	< 0.01	< 0.01
pH	7.41^a^	7.42^a^	7.38^b^	0.01	< 0.01	< 0.01	< 0.01

^1^*iCa*^*2*+^ Ion calcium, *pCO*_*2*_ Partial pressure of carbon dioxide, *sO*_*2*_ oxygen Saturation, *pO*_*2*_ Partial pressure of oxygen, *BEeff* Base excess extracellular fluid

^a–c^Means within the same row with different superscripts are different (*P* < 0.05)

Gas profiles in coccygeal artery of dairy cows are shown in Table S3. Under Mild-HS, the concentration of ionized Ca^2+^ was greater, whereas the concentration of K^+^ was lower than those under No-HS (*P* < 0.05). Under Mod-HS, greater K^+^ and lower total CO_2_, and BEecf (*P* < 0.05) were observed relative to those under No-HS and Mild-HS. No significant differences (*P*_treat_ > 0.05) were identified in the sO_2_ and pO_2_ among different HS conditions. Percentage of HCT and concentration of HGB (*P* < 0.05) were lower under Mod-HS than under No-HS. Blood gas parameters in mammary vein indicated similar change trend as in coccygeal artery of dairy cows under varying HS (Table S4).

Table 3 showed the concentration of oxygen in the blood vessels of dairy cows. Oxygen concentration was lower in the coccygeal arteries and mammary veins of dairy cows under Mod-HS than in those under No-HS (*P* < 0.05), but was not significantly different (*P*_*treat*_ = 0.60) in the coccygeal veins among three HS conditions. The mammary AVD in oxygen concentration did not differ between HS and No-HS treatments (*P*_*treat*_ = 0.77).

**Table 3:** Oxygen concentration in the coccygeal vein, coccygeal artery, and mammary vein of dairy cows under different heat stress

Item, mL/dL	Heat stress			SEM	*P*-value		
	No	Mild	Moderate		Treat	Linear	Quadratic
Coccygeal vein	7.54	7.77	7.19	0.41	0.60	0.54	0.42
Coccygeal artery	11.6^a^	11.2^ab^	10.8^b^	0.20	< 0.01	< 0.01	0.81
Mammary vein	9.58^a^	9.00^ab^	8.69^b^	0.28	< 0.01	< 0.01	0.51
Mammary AVD^1^	2.09	2.26	2.21	0.20	0.77	0.62	0.61

^1^*AVD* Arterio-venous difference

^a,b^Means within the same row with different superscripts are different (*P* < 0.05)

### Blood hematological parameters

Hematological parameters from coccygeal vein of dairy cows are presented in Table 4. The RBC count (*P*_*treat*_ < 0.01), HGB concentration (*P*_*treat*_ < 0.01) and mean corpuscular hemoglobin concentration (MCHC) (*P*_*treat*_ < 0.01) were lower, whereas mean corpuscular volume (MCV) was greater (*P*_*treat*_ < 0.01) under Mild and Mod-HS than under No-HS. Percentage of HCT (*P*_*treat*_ < 0.01) was lower under Mod-HS than under No-HS and Mild-HS. Under Mild-HS, platelet concentration was lower (*P* < 0.01), whereas neutrophil proportion was greater (*P* < 0.01) than that under No-HS and Mod-HS.

**Table 4:** Hematological parameters in coccygeal vein of dairy cows under different heat stress

Item^1^	Heat stress			SEM	*P*-value		
	No	Mild	Moderate		Treat	Linear	Quadratic
RBC, M/μL	6.05^a^	5.81^b^	5.37^c^	0.11	< 0.01	< 0.01	0.12
HCT, %	28.0^a^	28.0^a^	25.9^b^	0.44	< 0.01	< 0.01	< 0.01
HGB, g/L	101^a^	98.2^b^	91.8^c^	1.44	< 0.01	< 0.01	0.13
MCV, fL	46.4^b^	48.5^a^	48.3^a^	0.70	< 0.01	< 0.01	< 0.01
MCH, pg	16.8	16.9	17.1	0.26	0.03	0.01	0.87
MCHC, g/L	363^a^	350^b^	354^b^	1.4	< 0.01	< 0.01	< 0.01
MPV, fL	7.34	7.45	7.29	0.13	0.07	0.45	0.03
PLT, K/μL	463^a^	420^b^	466^a^	27.2	< 0.01	0.83	< 0.01
WBC, K/μL	16.1^a^	15.9^a^	13.5^b^	1.29	< 0.01	< 0.01	0.03
NEU, %	47.1^b^	51.1^a^	47.4^b^	2.69	0.04	0.88	0.01
LYM, %	43.6	40.2	44.7	2.91	0.01	0.50	< 0.01
MON, %	8.28	7.95	7.03	0.49	0.08	0.03	0.54
EOS, %	0.95	0.64	1.08	0.24	0.40	0.70	0.20
BASO, %	0.24	0.18	0.15	0.03	0.22	0.09	0.72

^1^*RBC* Red blood cell, *HCT* Hematocrit, *HGB* Hemoglobin, *MCV* Mean corpuscular volume, *MCH* Mean corpuscular hemoglobin, *MCHC* Mean corpuscular hemoglobin concentration, *MPV* Mean platelet volume, *PLT* Platelet, *WBC* White blood cell, *NEU* Neutrophil, *LYM* Lymphocyte, *MON* Monocyte, *EOS* Eosimophil, *BASO* Basophil

^a–c^Means within the same row with different superscripts are different (*P* < 0.05)

### Variables related with heat stress, hypoxia stress and oxidative stress

Variables related to HS, oxidative stress, and hypoxia at different HS levels are presented in Table 5. Under Mod-HS, coccygeal vein concentrations of HO-1, VEGF, HIF-1α, HSP70, HSP90, and total bilirubin as well as activity of MDA were greater (*P* < 0.05) than those under No-HS and Mild-HS, with no difference between No-HS and Mild-HS (*P* > 0.05). Concentration of HSF showed an increasing trend (*P*_*treat*_ = 0.06) under Mod-HS. The iNOS concentration decreased (*P*_*treat*_ < 0.01) under Mod-HS compared to that under No-HS and Mild-HS, with no difference between No-HS and Mild-HS (*P* > 0.05). Concentration of SOD was lower (*P* < 0.05) under Mod-HS than under No-HS and Mild-HS, with no difference between Mild-HS and Mod-HS (*P* > 0.05). During Mild-HS, the concentration of GSH-Px was greater (*P* < 0.05) than under No-HS and Mod-HS.

**Table 5:** Heat stress-related variables, oxidative stress variables, and hypoxia variables in coccygeal vein of dairy cows during different heat stress

Item^1^	Heat stress			SEM	*P*-value		
	No	Mild	Moderate		Treat	Linear	Quadratic
NO, μmol/L	5.43	7.19	6.78	0.74	0.32	0.28	0.28
iNOS, U/mL	15.0^a^	13.4^b^	12.3^b^	0.31	< 0.01	< 0.01	0.42
eNOS, ng/mL	5.00	4.67	4.91	0.21	0.50	0.77	0.26
HO-1, ng/mL	17.7^b^	18.6^b^	23.4^a^	0.93	< 0.01	< 0.01	0.09
VEGF, ng/L	263^b^	262^b^	313^a^	10.4	< 0.01	< 0.01	0.02
MDA, nmol/mL	1.29^b^	1.40^b^	2.18^a^	0.17	< 0.01	< 0.01	0.07
SOD, U/mL	171^a^	167^a^	152^b^	4.1	< 0.01	< 0.01	0.08
GSH-Px, U/mL	40.9^b^	55.9^a^	42.1^b^	2.09	< 0.01	0.71	< 0.01
T-AOC, mmol/L	0.32	0.34	0.31	0.01	0.04	0.34	0.02
HSF, ng/L	252	244	282	12.6	0.06	0.07	0.11
HIF-1α, ng/L	207^b^	206^b^	255^a^	10.9	< 0.01	< 0.01	0.01
HSP27, ng/mL	4.38^ab^	3.88^b^	4.46^a^	0.17	0.02	0.70	0.01
HSP70, ng/mL	5.12^b^	4.83^b^	6.21^a^	0.24	< 0.01	< 0.01	< 0.01
HSP90, ng/mL	19.2^b^	19.6^b^	22.8^a^	0.76	< 0.01	< 0.01	0.06
Total bilirubin, μmol/L	2.31^b^	2.06^b^	3.83^a^	0.12	< 0.01	< 0.01	< 0.01

^1^*NO* Nitric oxide, *iNOS* Inducible nitric oxide synthase, *eNOS* Endothelial nitric oxide synthase, *HO-1* Heme oxygenase 1, *VEGF* Vascular endothelial growth factor, *MDA* Malondialdehyde, *SOD* Superoxide dismutase, *GSH-Px* Glutathione peroxidase, *T-AOC* Total antioxidant capacity, *HSF* Heat shock transcription factor, *HIF-1α* Hypoxia inducible factor 1α, *HSP70* Heat shock protein70

^a–c^Means within the same row with different superscripts are different (*P* < 0.05)

### Correlation analysis between blood gas profile and oxidative stress

**Fig. 1:**
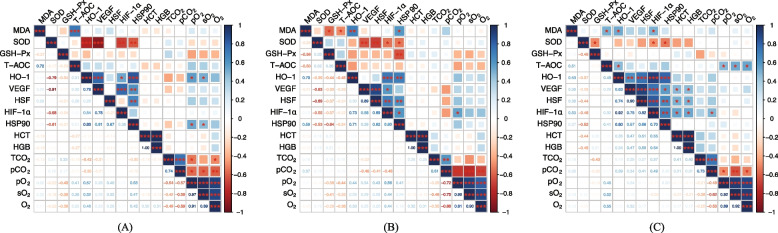
Pearson correlation analysis between blood gas profiles and oxidative stress in cow coccygeal veins under no heat stress (**A**), mild heat stress (**B**), and moderate heat stress (**C**). MDA, malondialdehyde; SOD, superoxide dismutase; GSH-Px, glutathione peroxidase; T-AOC, total antioxidant capacity; HO-1, heme oxygenase 1; VEGF, vascular endothelial growth factor; HSF, heat shock transcription factor; HIF-1α, hypoxia inducible factor 1α; HSP90, heat shock protein 90; HCT, hematocrit; HGB, hemoglobin; TCO_2_, total pressure of carbon dioxide, pCO_2_, partial pressure of carbon dioxide; pO_2_, partial pressure of oxygen, sO_2_, oxygen saturation; Color gradients indicate the degree of correlation, with blue indicating a positive correlation and red indicating a negative correlation. ^***^P < 0.001, ^**^P < 0.01, ^*^P < 0.05

Correlation coefficients between blood gas profiles and oxidative stress are shown in Fig. 1. Under No-HS, HSP90 was positively correlated with pO_2_ (*r* = 0.53, *P* = 0.02), whereas SOD was negatively correlated with HSP90 (*r* =  −0.61, *P* = 0.01) and HIF-1α (*r* =  −0.68, *P* < 0.01). Under Mild-HS, coccygeal vein concentrations of HIF-1α (*r* = 0.602, *P* = 0.011) and MDA (*r* = 0.60, *P* = 0.01) were positively correlated with HSP90 but negatively correlated with SOD (*r* =  −0.55, *P* = 0.02). The HSP90 was negatively correlated with SOD (*r* =  −0.53, *P* = 0.03) and GSH-Px (*r* =  −0.64, *P* < 0.01).

Under moderate HS, coccygeal vein concentration of HIF-1α showed a positive association with HSP90 (*r* = 0.70, *P* < 0.01), MDA (*r* = 0.50, *P* = 0.04), HCT *(r* = 0.55, *P* = 0.02), and HGB (*r* = 0.55, *P* = 0.02). Both HIF-1α (*r* =  −0.48, *P* = 0.05) and HSP90 (*r* =  −0.52, *P* = 0.03) were negatively correlated with SOD levels. The T-AOC was positively correlated with sO_2_ (*r* = 0.52, *P* = 0.03) and oxygen content (*r* = 0.55, *P* = 0.02).

## Discussion

Heat stress is known to reduce feed intake and hence lactation performance [3], cause oxidative stress [7], and change substance metabolism in dairy cows [13]. However, our study primarily focused on exploring the additional metabolic alterations induced by HS, excluding the effects attributable to diminished feed intake. The role of oxygen metabolism in substance metabolism has seldom been reported. In this study, we measured milk composition, blood gas profiles, and oxygen concentration to estimate oxygen availability, and metabolites related to oxidative stress and hypoxia stress in dairy cows under HS with an aim to explore how HS affects dairy cow physiology, health and lactation performance from the perspective of oxygen metabolism. As such, we hypothesize that the observed variations in oxygen concentration may be attributable to changes in metabolic activity under HS, particularly in tissues with high oxygen demand such as the mammary gland.

Our findings demonstrate a significant decline in milk yield and milk fat percentage influenced by HS. We also observed an augmentation in the count of milk somatic cells and a substantial alteration in the immune cells and in the blood parameters of the coccygeal vein. Increased milk somatic cells are associated with immune activation, and excessive immune system activation under HS can use the increased amount of glucose, thereby reducing their availability for lactose synthesis and reducing milk production [27]. Increased sweating and respiratory alkalosis during HS can affect the homeostasis of electrolytes in blood [28]. Therefore, the changes of Ca^2+^, Na^+^ and K^+^ under different degrees of HS may indicate the changes in electrolyte balance and body health of dairy cows. Electrolyte concentrations in milk may reveal short-term physiological responses related to HS [29]. These results suggest that HS exerts a notable impact on the overall health status of dairy cows. Furthermore, during HS (with an increase in respiratory rate) dairy cows undergo intense gas exchange with the external environment, including ion and acid–base balance in the coccygeal vein and coccygeal artery. Fluctuations in temperature and humidity throughout the day can lead to variations in cow blood parameters [16]. Given our focus on identifying indicators of consistent and stable changes during HS, we restricted our blood sample collection to the morning. However, this approach imposes certain limitations on analyzing the dynamics of cow blood parameters. To gain a comprehensive understanding of the impact of HS on the physiological health of dairy cows, future research should consider the broader spectrum of blood biochemical changes in relation to diurnal fluctuations of temperature and humidity.

Contrary to expectations, our study did not reveal any alterations in pO_2_ and sO_2_ in both coccygeal vein and coccygeal artery, implying that the amount of oxygen circulating in the blood in dairy cows may remain relatively unaltered in response to sustained HS [30]. In addition, no significant difference was found in the mammary AVD of oxygen concentration among different HS, which may be attributed to adaptation of dairy cows to the environment because the oxygen concentration indicated parallel changes in coccygeal artery and mammary vein. Notably, our findings indicated a decline in the counts of RBC, along with a decreased concentration of HGB and MCHC in the coccygeal vein during HS conditions. The HGB is an iron-containing protein complex that transports oxygen throughout the body and is usually found in RBC [31]. Arterial oxygen concentration and consequently tissue oxygenation are directly affected by HGB levels [32, 33]. Both HCT and HGB have been considered as essential indices reflecting the capacity of RBCs to carry oxygen [34]. Lower level of HCT and lower count of RBC were found in Holstein dairy cows during the summer than during the winter [14], consistent with the results of our study. Morar et al. [35] reported that RBC count and levels of HCT and HGB were significantly lower in cows under HS. In our study, oxygen concentrations in both coccygeal artery and mammary vein exhibited a substantial decrease during periods of Mod-HS. This trend correlated with a reduction in the count of RBC and the concentration of HGB. The observed data hint at a potential physiological response to HS in cows, where the availability of oxygen is modulated via alterations in RBC count and HGB concentration. The precise mechanisms underlying this adaptation, however, warrant further in-depth investigations.

Our study furnishes compelling evidence suggesting a significant diminution in the MCHC, consistent with the change in HGB during periods of HS. Bilirubin is a naturally occurring product from the catabolism of heme by heme oxygenase [36, 37]. Serum levels of bilirubin are thought to be derived from the breakdown of RBC, which mostly occurs in the spleen [38]. Our findings elucidate a significant elevation in total bilirubin concentration during the period of Mod-HS. This increase may be attributable to an augmentation in the breakdown of RBC, consequently resulting in a diminished RBC count [39]. Concurrently, this process could lead to the liberation of hemoglobin within RBCs and subsequent catalysis into bilirubin by HO-1, which was enhanced by HS.

Beyond the reduction in RBC, HS incurs broader physiological implications, influencing overall health. The HS leads to excessive ROS production and mitochondrial dysfunction, resulting in oxidative stress in dairy cows [7]. Biological macromolecules are easily damaged by oxidative stress, which interferes with metabolic and physiological pathways [40]. In the current study, the concentrations of MDA increased and SOD decreased in the coccygeal vein of dairy cows during HS. The MDA is the main product of polyunsaturated fatty acid peroxidation. The increased MDA concentration may be attributable to the enhanced production of ROS and aggravating the oxidative damage of lipids [41, 42]. The SOD catalyzes the disproportionation of superoxide anion radicals (O_2_^−^) into H_2_O_2_ and O_2_. Therefore, the observed elevation of GSH-Px during Mild-HS may conceivably enhance the antioxidant capacity of dairy cows. However, during the Mod-HS, the elevated levels of MDA alongside the decreased SOD within the coccygeal veins suggest that the cows may be subjected to intense oxidative stress.

The oxidative stress induced by HS appears to have a significant correlation with oxygen metabolism, as our findings suggest. Mitochondria are the primary sites of oxygen consumption and ROS production, accounting for 85%–90% of cell oxygen consumption [43, 44]. The mitochondrial electron transport chain requires molecular oxygen to produce ATP [45]. The HS has been reported to cause mitochondrial protein degeneration and inhibit mitochondrial ATP synthesis [46]. A decrease in oxygen consumption suggests damage to the mitochondrial respiratory chain under HS [47]. It has been shown that HS inactivated complex I in the respiratory chain [48]. Reduced electron flow along the respiratory chain leads to decreased oxygen uptake and increased mitochondrial superoxide anion levels [49], which are precursors of most ROS and mediators of oxidation chain reactions. Previous studies have shown that HS can reduce *SOD-1* mRNA levels, cytoplasmic SOD protein levels, and enzyme activity by increasing ROS [50]. Therefore, it seems that the mechanism by which HS impacts oxygen availability may be associated with the oxidative stress resulting from mitochondrial degradation.

In the subsequent correlation analysis, we found that HIF-1α and HSP90 were negatively correlated with SOD during HS, indicating that cows with higher HIF-1α and HSP90 levels might have a weaker oxidative resistance. A significant positive correlation existed between HIF-1α, MDA, and HSP90 under Mild-HS, indicating that cows with greater HSP90 levels during HS may be a potential risk for hypoxia stress and lipid peroxidation. In order to dissipate body heat under HS, animals experience an increased peripheral vascular dilation and a compensatory decrease in intestinal blood flow, resulting in hypoxia [27]. Therefore, HS led to both hypoxia and oxidative stress, which affected the health of dairy cows in our study. The HIF-1α appears to be a master transcription factor capable of inducing the expression of genes related to autoregulation, cell survival and proliferation, angiogenesis, energy metabolism, and erythropoiesis [51], and is induced by cellular responses to hypoxia. Hypoxia transactivates target genes such as VEGF [52] and directly enhances angiogenesis by promoting VEGF expression [53]. The VEGF plays a crucial role in the hypoxia response by controlling the expression of many hypoxia response genes involved in various oxygen delivery processes [54]. Our investigation revealed that during Mod-HS there is a pronounced increase in VEGF concentrations, suggesting that HIF-1α, in response to HS, stimulates the expression of VEGF, thereby fostering angiogenesis. The increased expression of HSP90 and HIF-1α in dairy cows during HS increased VEGF expression and stimulates angiogenesis, which may play a specific role in the adaptation of dairy cows to HS. Therefore, cows under HS self-regulated to improve oxygen-carrying capacity; however, their oxygen transport and hematopoietic functions were still deteriorated to some extent.

It should be pointed out that gradual adaptation to HS may exist, wherein dairy cows exhibit adaptive responses when subjected to mild to moderate HS. In our experiment, we were unable to account for the potential influence of time on this adaptation process. It is plausible that cows gradually acclimate to HS from No-HS condition to Mild-HS condition before being exposed to Mod-HS condition. On the other hand, a recent study showed that 7 d of washout was enough for recovery in milk yield, and inflammatory markers after a period of HS [27]. Therefore, it is important to incorporate a thermoneutral control group in future experiments to enhance experimental design and better assess the effects of HS.

## Conclusion

In this study, heat stressed-dairy cows had greater concentrations of HIF-1α and MDA in the coccygeal vein, but lower concentrations of oxygen, HGB, and RBC in the blood vessels than the cows under No-HS. These findings imply that the exposure to HS decreases the availability of circulating oxygen by reducing hemoglobin concentration and RBC count, thus instigating oxidative stress and hypoxia. Therefore, HS impacted lactation performance by affecting the ability of the blood to metabolize and transport oxygen. However, the precise mechanism underlying how HS influences mammary lactation via the availability of blood oxygen necessitates further exploration.

### Supplementary Information

**Additional file 1: Table S1.** Basal diet ingredients and chemical composition. **Table S2.** Rectal temperature and respiration rate of dairy cows at three different time points under varying heat stress. **Table S3.** Blood gas parameters in coccygeal artery of dairy cows under varying heat stress. **Table S4.** Blood gas parameters in mammary vein of dairy cows under varying heat stress. **Fig. S1.** Farm environmental parameters during the experiment and heat stress (HS) effects on the physiological variables of dairy cows. **a** Changes in average temperature, humidity, and temperature-humidity index (THI) during the experimental period. **b** The sampling day, variables and experiment time line under No-HS with THI below 68 (from May 14 to May 21), Mild-HS (68 ≤ THI ≤ 79, from May 22 to June 18), and Moderate-HS (79 < THI ≤ 88, from June 21 to July 14), respectively. **c** The THI on the sampling day under No-HS (May 15), Mild-HS (June 18), and Moderate-HS (July 14) , respectively.

## Data Availability

All data generated or analyzed during this study are included in the published article.

## Abbreviations

BEecf: Base excess in the extracellular fluid
ECM: Energy-corrected milk yield
eNOS: Endothelial nitric oxide synthase
FCM: Fat corrected milk yield
GSH-Px: Glutathione peroxidase
HCT: Hematocrit
HGB: Hemoglobin
HIF-1α: Hypoxia inducible factor 1α
HO-1: Heme oxygenase 1
HSF: Heat shock transcription factor
HSP70: Heat shock protein 70
iCa^2+^: Ionized calcium
iNOS: Inducible nitric oxide synthase
MCV: Mean corpuscular volume
MCH: Mean corpuscular hemoglobin
MCHC: Mean corpuscular hemoglobin concentration
MDA: Malondialdehyde
Mild-HS: Mild heat stress
Mod-HS: Moderate heat stress
MPV: Mean platelet volume
MUN: Milk urea nitrogen
NO: Nitric oxide
No-HS: No heat stress
pCO_2_: Partial pressure of carbon dioxide
pO_2_: Partial pressure of oxygen
RBC: Red blood cell
RH: Relative humidity
RR: Respiratory rate
RT: Rectal temperatures
SCC: Somatic cell count
sO_2_: Oxygen saturation
SOD: Superoxide dismutase
T-AOC: Total antioxidant capacity
THI: Temperature-humidity index
VEGF: Vascular endothelial growth factor
WBC: White blood cell
