# Supplementary material for: Heat stress affects dairy cow health status through blood oxygen availability
Source: J Anim Sci Biotechnol. 2023 Sep 2;14:112. doi: 10.1186/s40104-023-00915-3 (PMC10474781; doi:10.1186/s40104-023-00915-3)
Supplement: Supplementary file 1 — Additional file 1: Table S1. Basal diet ingredients and chemical composition. Table S2. Rectal temperature and respiration rate of dairy cows at three different time points under varying heat stress. Table S3. Blood gas parameters in coccygeal artery of dairy cows under varying heat stress. Table S4. Blood gas parameters in mammary vein of dairy cows under varying heat stress. Fig. S1. Farm environmental parameters during the experiment and heat stress (HS) effects on the physiological variables of dairy cows. a Changes in average temperature, humidity, and temperature-humidity index (THI) during the experimental period. b The sampling day, variables and experiment time line under No-HS with THI below 68 (from May 14 to May 21), Mild-HS (68 ≤ THI ≤ 79, from May 22 to June 18), and Moderate-HS (79 < THI ≤ 88, from June 21 to July 14), respectively. c The THI on the sampling day under No-HS (May 15), Mild-HS (June 18), and Moderate-HS (July 14) , respectively. [file 40104_2023_915_MOESM1_ESM.docx]

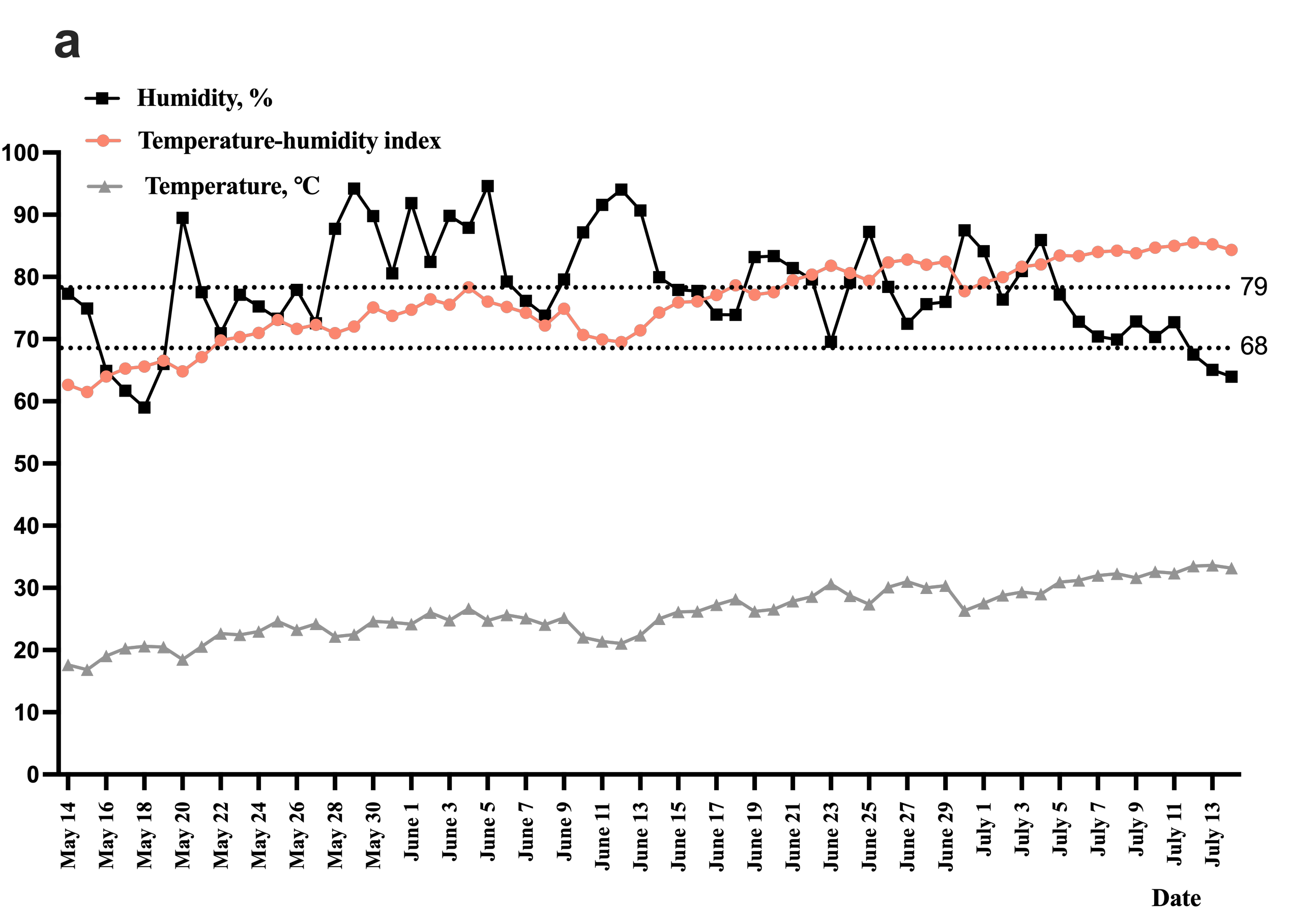


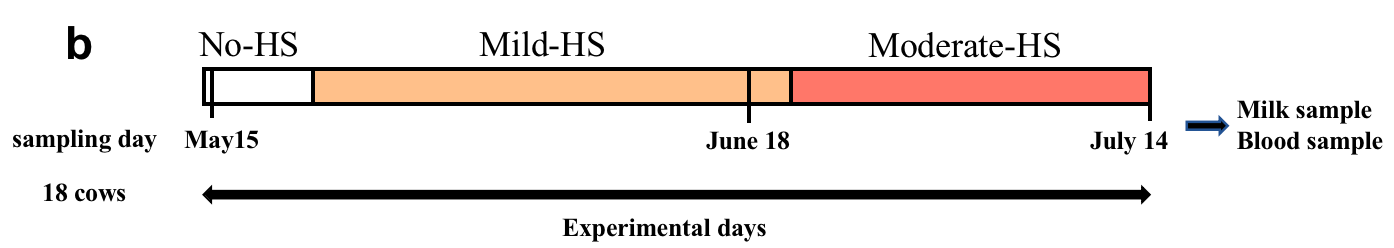


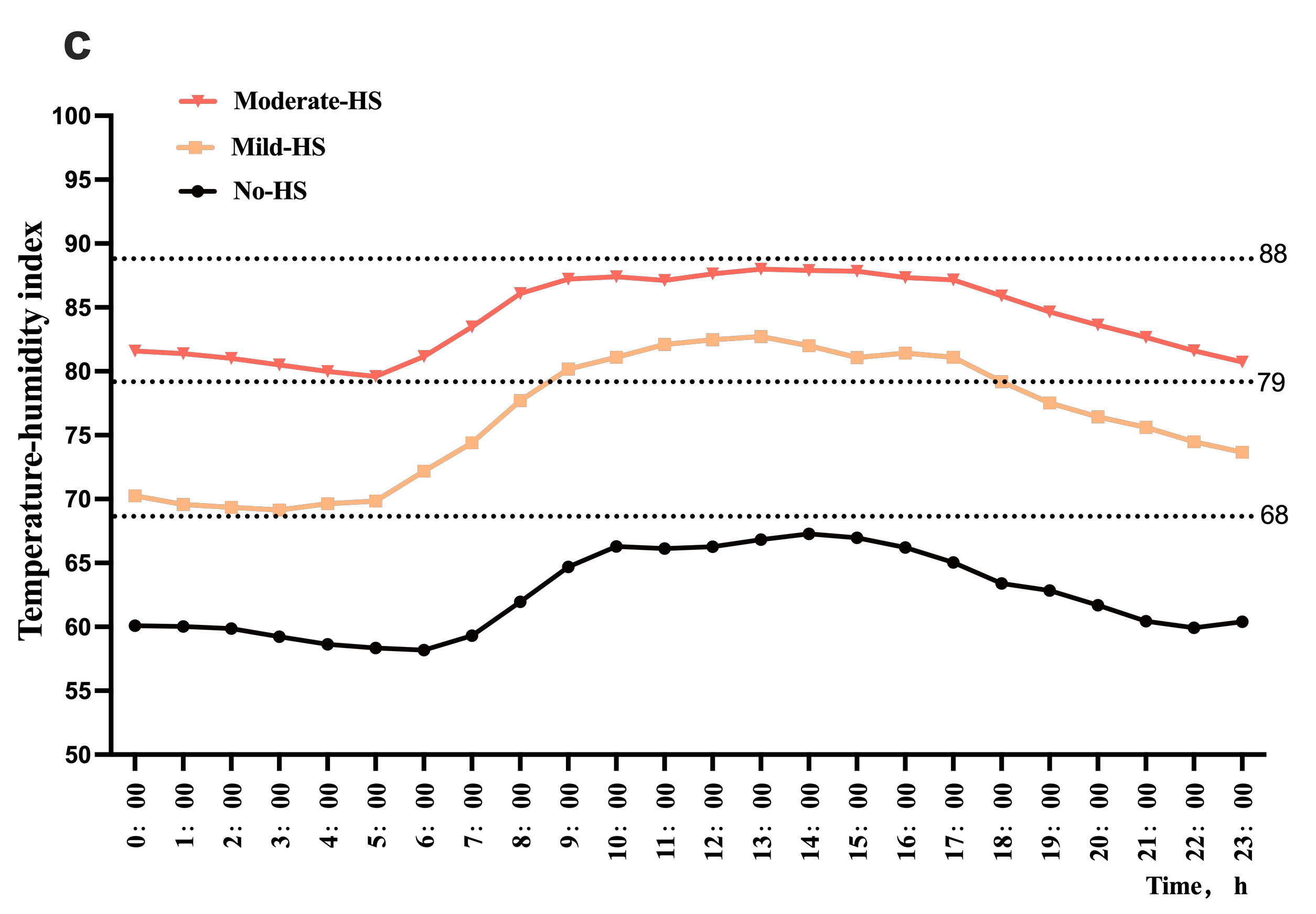


**Fig. S1** Environmental parameters on the farm during the experiment period with different heat stress (HS). **a** Changes in the average temperature, humidity, and temperature-humidity index (THI). **b** The sampling day, variables and experiment time line under No-HS with THI below 68 (from May 14 to May 21), Mild-HS (68 ≤ THI ≤ 79, from May 22 to June 18), and Moderate-HS (79 < THI ≤ 88, from June 21 to July 14), respectively. **c** The THI on the sampling day under No-HS (May 15), Mild-HS (June 18), and Moderate-HS (July 14), respectively

**Table S1** Ingredients and chemical compositions of the basal diet

| **Ingredients** | **% (DM basis)** | | **Composition** | **% of DM** |
| --- | --- | --- | --- | --- |
| Oat hay | | 13.51 | Crude protein | 17.9 |
| Alfalfa hay | | 8.48 | Neutral detergent fiber | 34.0 |
| Corn silage | | 19.27 | Acid detergent | 23.2 |
| Cottonseed meal | | 4.89 | Non-fiber carbohydrate | 36.1 |
| Brewer's grains | | 4.02 | Starch | 24.5 |
| Soy sauce residue | | 0.89 | Fat | 4.4 |
| Beet pulp | | 5.71 | Ash | 9.3 |
| Ground corn | | 20.97 | Ca | 0.74 |
| Rapeseed meal | | 1.34 | P | 0.56 |
| Soybean meal | | 11.60 | Mg | 0.34 |
| Extruded soybean | | 2.83 | K | 1.50 |
| Molasses | | 1.29 | NE_L_2, MJ/kg DM | 6.61 |
| Fat meal | | 0.87 |  |  |
| CaHPO_4_ | | 0.31 |  |  |
| NaCl | | 0.48 |  |  |
| Limestone | | 0.86 |  |  |
| NaHCO_3_ | | 0.86 |  |  |
| MgO | | 0.29 |  |  |
| Premix1 | | 1.53 |  |  |
| ^1^ Formulated to provide (per kg of DM): vitamin A 600 KIU, vitamin D3 150 KIU, vitamin E 2,000 IU, nicotinic acid 500 mg, Cu 1,500 mg, Fe 1,500 mg, Mn 1,500 mg, Zn 7,000 mg, I 90 mg, Se 50 mg, Co 20 mg | | | | |
| 2 NE_L_= Net energy for lactation | | | | |

**Table S2** Rectal temperature and respiration rate of dairy cows at three different time points under varying heat stress

| **Item^1^** | **Time, h** | **Heat stress** | | |
| --- | --- | --- | --- | --- |
|  |  | **No** | **Mild** | **Moderate** |
| Respiratory rate, bpm | 0800 | 41.85±6.95 | 43.97±9.71 | 75.83±14.19 |
|  | 1400 | 44.33±8.74 | 60.81±12.31 | 83.69±12.98 |
|  | 2000 | 43.68±6.76 | 42.69±9.67 | 69.86±10.35 |
| Rectal temperature, ℃ | 0800 | 38.34±0.18 | 38.48±0.20 | 39.47±0.34 |
|  | 1400 | 38.42±0.23 | 39.05±0.29 | 39.98±0.56 |
|  | 2000 | 41.85±6.95 | 43.97±9.71 | 39.56±0.39 |

^1^ Rectal temperature and respiration rate were measured at 0800, 1400, and 2000 in the morning, noon, and evening, respectively. Data are presented as mean ± standard deviation (SD)

**Table S3** Blood gas parameters in coccygeal artery of dairy cows under varying heat stress

| **Item^1^** | **Heat stress** | | | **SEM** | ***P*-value** | | |
| --- | --- | --- | --- | --- | --- | --- | --- |
|  | **No** | **Mild** | **Moderate** |  | **Treat** | **Linear** | **Quadratic** |
| iCa^2+^, mmol/L | 1.18^b^ | 1.99^a^ | 1.18^b^ | 0.06 | <0.01 | 0.99 | <0.01 |
| Na^+^, mmol/L | 139 | 134 | 138 | 1.64 | 0.06 | 0.61 | 0.02 |
| K^+^, mmol/L | 4.07^a^ | 3.84^b^ | 3.83^b^ | 0.05 | <0.01 | <0.01 | 0.07 |
| pO_2_, mmHg | 104 | 114 | 115 | 5.09 | 0.25 | 0.12 | 0.55 |
| sO_2_, % | 97.9 | 98.7 | 98.2 | 0.33 | 0.30 | 0.53 | 0.16 |
| Total CO_2_, mmol/L | 29.3^a^ | 30.1^a^ | 25.9^b^ | 0.60 | <0.01 | <0.01 | <0.01 |
| pCO_2_, mmHg | 38.4^ab^ | 38.2^a^ | 35.9^b^ | 0.77 | 0.02 | 0.01 | 0.21 |
| HCO_3_^-^, mmol/L | 28.1^a^ | 28.9^a^ | 24.8^b^ | 0.58 | <0.01 | <0.01 | <0.01 |
| BEecf, mmol/L | 4.44^a^ | 5.83^a^ | 0.82^b^ | 0.65 | <0.01 | <0.01 | <0.01 |
| pH | 7.47^ab^ | 7.49^a^ | 7.45^b^ | 0.01 | <0.01 | 0.03 | <0.01 |

^1^ iCa^2+^, ion calcium; pCO_2_, partial pressure of carbon dioxide; sO2, oxygen saturation; pO_2_, partial pressure of oxygen; BEeff, base excess extracellular fluid; HCT, hematocrit; HGB, hemoglobin

^a–c^ Means in the same row with different superscripts are different (*P* < 0.05)

**Table S4** Blood gas parameters in mammary vein of dairy cows under varying heat stress

| **Item^1^** | **Heat stress** | | | **SEM** | ***P*-value** | | |
| --- | --- | --- | --- | --- | --- | --- | --- |
|  | **No** | **Mild** | **Moderate** |  | **Treat** | **Linear** | **Quadratic** |
| iCa^2+^, mmol/L | 1.15^b^ | 1.86^a^ | 1.15^b^ | 0.05 | <0.01 | 0.99 | <0.01 |
| Na^+^, mmol/L | 138^b^ | 135^c^ | 139^a^ | 0.40 | <0.01 | <0.01 | <0.01 |
| K^+^, mmol/L | 3.99 | 3.76 | 3.86 | 0.05 | <0.01 | 0.02 | <0.01 |
| pO_2_, mmHg | 41.8 | 42.7 | 41.5 | 1.42 | 0.77 | 0.87 | 0.49 |
| sO_2_, % | 79.9 | 80.0 | 79.0 | 1.52 | 0.81 | 0.60 | 0.70 |
| Total CO_2_, mmol/L | 30.8^a^ | 31.5^a^ | 26.9^b^ | 0.61 | <0.01 | <0.01 | <0.01 |
| pCO_2_, mmHg | 40.3 | 40.4 | 38.6 | 0.73 | 0.11 | 0.07 | 0.27 |
| HCO_3_^-^, mmol/L | 29.6^a^ | 30.2^a^ | 25.7^b^ | 0.58 | <0.01 | <0.01 | <0.01 |
| BEecf, mmol/L | 6.06^a^ | 6.76^a^ | 1.41^b^ | 0.66 | <0.01 | <0.01 | <0.01 |
| pH | 7.47^a^ | 7.48^a^ | 7.43^b^ | 0.01 | <0.01 | <0.01 | <0.01 |

^1^ iCa^2+^, ion calcium; pCO_2_, partial pressure of carbon dioxide; sO2, oxygen saturation; pO_2_, partial pressure of oxygen; BEeff, base excess extracellular fluid

^a–c^ Means in the same row with different superscripts are different (*P* < 0.05)
